# Lgals3bp suppresses colon inflammation and tumorigenesis through the downregulation of TAK1-NF-κB signaling

**DOI:** 10.1038/s41420-021-00447-7

**Published:** 2021-04-06

**Authors:** Sang-Hee Cho, Hyun-Jeong Shim, Mi-Ra Park, Ji-Na Choi, Md Rashedunnabi Akanda, Jun-Eul Hwang, Woo-Kyun Bae, Kyung-Hwa Lee, Eun-Gene Sun, Ik-Joo Chung

**Affiliations:** 1 Department of Hematology and Oncology, Chonnam National University Medical School and Hwasun Hospital, Hwasun, Republic of Korea; 2 Immunotherapy Innovation Center, Chonnam National University Medical School and Hwasun Hospital, Hwasun, Republic of Korea; 3 Combinatorial Tumor Immunotherapy MRC Center, Chonnam National University Medical School, Hwasun, Republic of Korea; 4 Department of Pathology, Chonnam National University Medical School and Hwasun Hospital, Hwasun, Republic of Korea; 5 Department of Pharmacology and Toxicology, Sylhet Agricultural University, Sylhet, Bangladesh

**Keywords:** Colorectal cancer, Inflammation

## Abstract

Galectin 3-binding protein (LGALS3BP, also known as 90K) is a multifunctional glycoprotein involved in immunity and cancer. However, its precise role in colon inflammation and tumorigenesis remains unclear. Here, we showed that *Lgals3bp*^−/−^ mice were highly susceptible to colitis and colon tumorigenesis, accompanied by the induction of inflammatory responses. In acute colitis, NF-κB was highly activated in the colon of *Lgals3bp*^−/−^ mice, leading to the excessive production of pro-inflammatory cytokines, such as IL-6, TNFα, and IL-1β. Mechanistically, Lgals3bp suppressed NF-κB through the downregulation of TAK1 in colon epithelial cells. There was no significant difference in the pro-inflammatory cytokine levels between wild-type and *Lgals3bp*^−/−^ mice in a chronic inflammatory state, during colon tumorigenesis. Instead, *Lgals3bp*^−/−^ mice showed elevated levels of GM-CSF, compared to those in WT mice. We also found that GM-CSF promoted the accumulation of myeloid-derived suppressor cells and ultimately increased colon tumorigenesis in *Lgals3bp*^−/−^ mice. Taken together, Lgals3bp plays a critical role in the suppression of colitis and colon tumorigenesis through the downregulation of the TAK1-NF-κB-cytokine axis. These findings suggest that LGALS3BP is a novel immunotherapeutic target for colon inflammation and tumorigenesis.

## Introduction

Inflammatory bowel disease (IBD) is an inflammatory disorder of the gastrointestinal tract. Excessive, chronic inflammation promotes colorectal cancer (CRC) development. Therefore, IBD patients are at high risk of CRC development^1,2^. CRC is the fourth most lethal malignancy worldwide^3^. Overall, cancer therapies, such as surgery, radiotherapy, and chemotherapy, fail to restrict CRC metastasis^4^. Therefore, it is necessary to further elucidate the molecular mechanisms underlying IBD pathogenesis and CRC progression.

NF-κB is a critical regulator of inflammation, cancer, and immunity^5,6^. Many cellular stimuli activate the NF-κB pathway through transforming growth factor-β-activated kinase 1 (TAK1)^7^. TAK1 was originally identified as a transforming growth factor-β (TGF-β)-activated mitogen-activated protein kinase kinase kinase^8^. However, it was later characterized and widely accepted as a key player in inducing pro-inflammatory cytokine signals, including tumor necrosis factor-α (TNF-α), interleukin-1 (IL-1), and TLR ligands^9^. Because TNF-α and IL-1 not only stimulate TAK1-NF-κB signaling but also target genes, positive feedback loops are induced in a paracrine or autocrine manner^10^. Hyper-activated TAK1 signaling causes severe inflammatory disorders and inflammation-associated cancer^9^. Thus, targeting TAK1 is a potential therapeutic approach for colon inflammation and tumorigenesis.

Galectin-3-binding protein, LGALS3BP, is a multifunctional secreted glycoprotein involved in immunity, inflammation, and cancer^11,12^. LGALS3BP was shown to downregulate the secretion of IL-4, IL-5, and IL-13 and to activate antiviral responses^13,14^. Previously, we have shown that Lgals3bp inhibits NF-κB inflammatory signaling pathways via TAK1 dephosphorylation and degradation in macrophages and mouse embryonic fibroblasts^15^. Because Lgals3bp expression is increased in cancer patients, its role in cancer has been widely studied^16–22^. Although the current evidence regarding Lgals3bp activity in cancer is controversial, Lgals3bp exhibits anti-tumor activity in CRC cells. It suppresses Wnt signals via the ISGylation-dependent ubiquitination of beta-catenin, but is downregulated in advanced CRC tissues^19^. Furthermore, CRC patients exhibiting high Lgals3bp expression in cancer tissues have a lower relapse risk and longer overall survival than those exhibiting low Lgals3bp expression^21^. Therefore, Lgals3bp induction is a therapeutic option for colon cancer. Despite the findings regarding the functions of Lgals3bp in colon cancer, its precise role in colon inflammation and tumorigenesis is unknown. In CRC tumor microenvironments (TMEs), myeloid-derived suppressor cells (MDSCs) promote cancer progression by suppressing T cells. Various factors are involved in MDSC differentiation and infiltration. Granulocyte-macrophage colony-stimulating factor (GM-CSF) and granulocyte colony-stimulating factor (G-CSF) are involved in MDSC accumulation during colon tumorigenesis^23,24^. Galectin-3, the Lgals3bp-binding protein, induces the recruitment of MDSCs into the Lewis lung cancer TME, in response to cisplatin^25^. However, the roles of Lgals3bp in MDSC expansion and TME remain unexplored. Thus, it is important to explore the roles of Lgals3bp in colon inflammation, tumorigenesis, and TME.

In this study, we investigated the roles of Lgals3bp in colonic inflammation and tumorigenesis using mouse models.

## Results

### Lgals3bp expression is significantly increased in the colon of mice with DSS-induced colitis

**Fig. 1:**
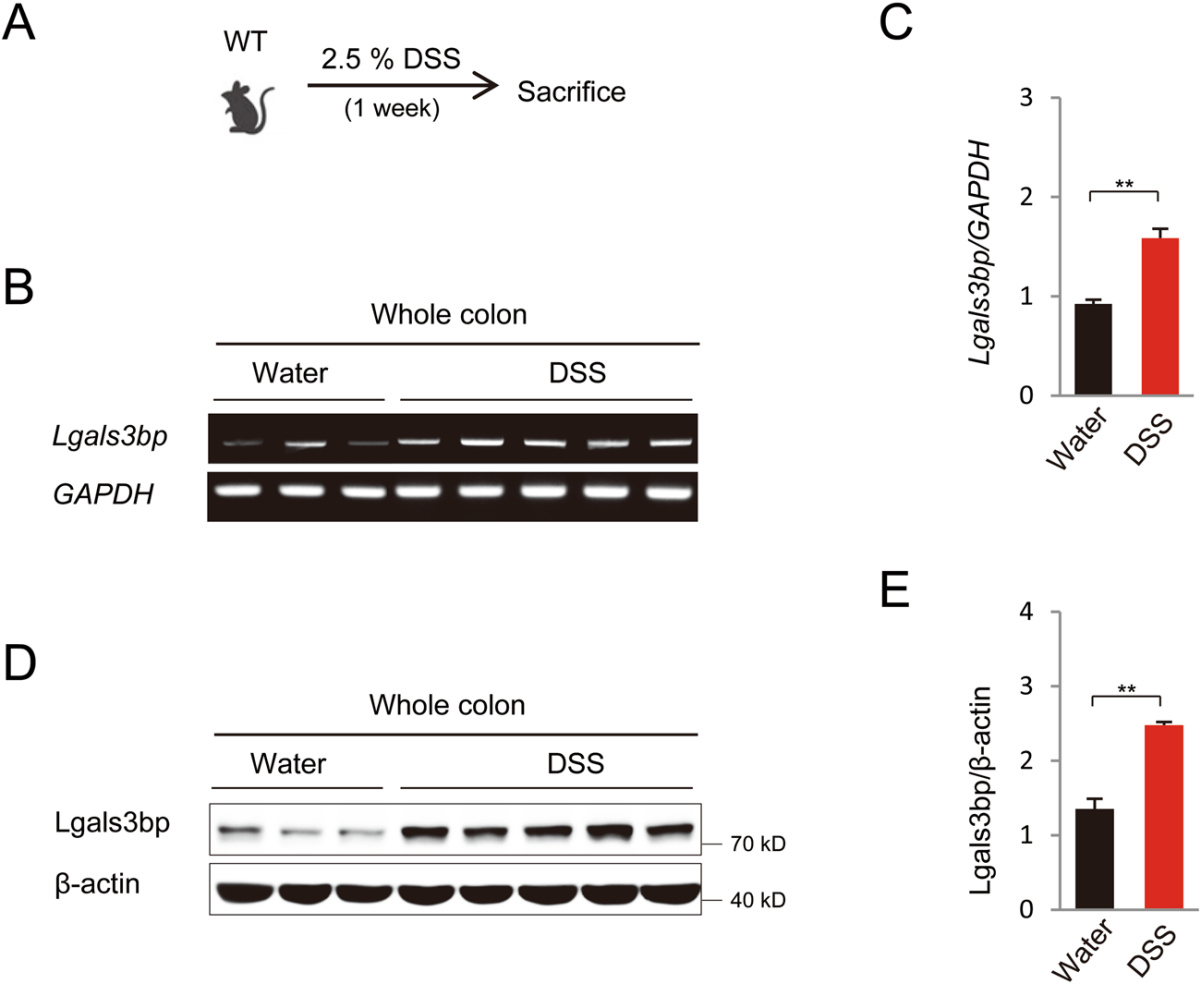
Elevated Lgals3bp expression in the colon of DSS-induced colitis mice. **A** Scheme of DSS-induced colitis in WT mice. Mice (*n* = 5) were administrated 2.5% DSS in drinking water for 7 days and sacrificed on day 7. Water­fed mice (*n* = 3) served as control; colon tissue was collected. **B**, **C** RNA was isolated from whole colons, and *Lgals3bp* and *GAPDH* expression was analyzed using RT-PCR (**B**) and RT-qPCR (**C**). **D** Protein extracts of the whole colon from WT and *Lgals3bp*^−/−^ mice were analyzed for the expression of Lgals3bp and β-actin proteins via western blotting. **E** Densitometric analysis of band intensities of western blots is shown in (**D**). Data are presented as means ± SEM values. **p < 0.01; ***p < 0.001.

RT-qPCR analysis revealed that *Lgals3bp* mRNA was ubiquitously expressed in various tissues, including the colon, spleen, liver, and lymph nodes (Fig. S1a). The colon is composed of an epithelial layer and the lamina propria, which is the connective tissue underlying the epithelium. These two fractions were analyzed to further determine the source of Lgals3bp expression in the colon. Western blotting analysis showed that Lgals3bp was expressed on both epithelial cells and the lamina propria (Fig. S1e). Furthermore, Lgals3bp mRNA and protein levels were significantly increased in the colons of DSS-treated mice, as compared to those in the water-fed group (Fig. 1A–E).

### Lgals3bp deficiency promotes colitis in mice

**Fig. 2:**
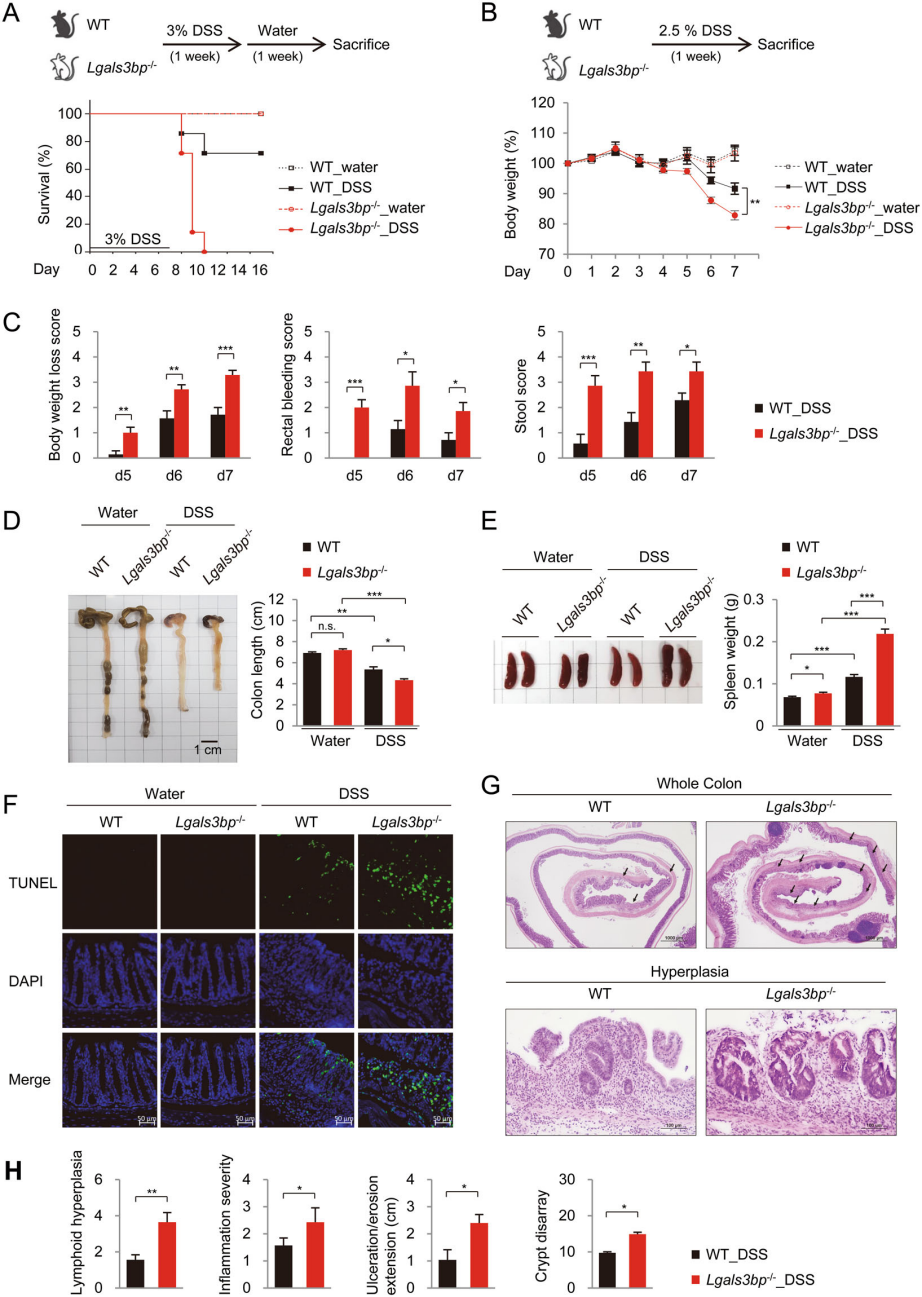
Hyper-susceptibility of *Lgals3bp*^−/−^ mice to DSS-induced colitis. **A** Relative survival rate. WT (*n* = 7) or *Lgals3bp*^−/−^ (*n* = 7) mice were orally administrated 3% DSS in drinking water for 7 days, followed by regular drinking water. Survival was monitored until day 15 after the start of DSS administration. WT (*n* = 4) or *Lgals3bp*^−/−^ (*n* = 4) mice provided with water served as controls. **B**–**E** WT (*n* = 7) and *Lgals3bp*^−/−^ (*n* = 7) mice were fed 2.5% DSS for 7 days. **B** Body weight change and (**C**) disease activity index of mice while administering 2.5% DSS in drinking water. **D** Colon length and (**E**) spleen weight measurement at day 7 after 2.5% DSS administration. (F-H) WT and *Lgals3bp*^−/−^ mice were fed 2.5% DSS for 7 days, followed by regular drinking water for 7 days. **F** Fluorescent microscopy images of TUNEL stained apoptotic cells in paraffin sections of mouse colons counterstained with DAPI. **G** Upper: Representative pictures of H&E-stained colon tissue sections. Arrow indicates histopathological changes. Lower: Representative dysplasia in the colon. **H** Histological analysis of colon tissue. H&E stained sections were scored as described in Materials and Methods. Data are presented as means ± SEM. *p < 0.05; **p < 0.01; ***p < 0.001; ****p < 0.0001.

To investigate the potential role of Lgals3bp in colon inflammation, we generated *Lgals3bp*^−/−^ mice. Gene knockout was evaluated by Sanger sequencing (Fig. S1b) and confirmed in the colon and spleen by RT-PCR and western blotting (Fig. S1c, d). First, we challenged wild-type (WT) and *Lgals3bp*^−/−^ mice with 3% DSS in drinking water for 7 days, to determine their lethality rate. In comparison with WT mice, *Lgals3bp*^−/−^ mice exhibited a significantly higher mortality (Fig. 2A). To establish the experimental colitis model, mice were treated with 2.5% DSS for 7 days and analyzed for colitis severity. *Lgals3bp*^−/−^ mice exhibited significantly greater body weight loss, rectal bleeding, and diarrhea than WT mice (Fig. 2B, C) and a significant shortening of the colon and splenomegaly (Fig. 2D, E), which reflects colonic and systemic inflammation, respectively. These results indicated that *Lgals3bp*^−/−^ mice were more prone to develop severe colitis than WT mice. Because the colon tissue was severely damaged in DSS-treated mice (data not shown), animals were allowed to recover for 1 week after DSS feeding, prior to tissue damage analysis. TUNEL assays showed that colonic apoptosis was increased in *Lgals3bp*^−/−^ mice, as compared to that in WT mice (Fig. 2F). Colon tissue alterations were further assessed by histopathological analysis. Histology scores for lymphoid hyperplasia, severity of inflammation, ulceration, and crypt disarray were significantly higher in *Lgals3bp*^−/−^ mice than in WT mice (Fig. 2G, H). Overall, *Lgals3bp*^−/−^ mice were highly susceptible to colitis.

### Lgals3bp deficiencies induce inflammatory responses in the colon during colitis

**Fig. 3:**
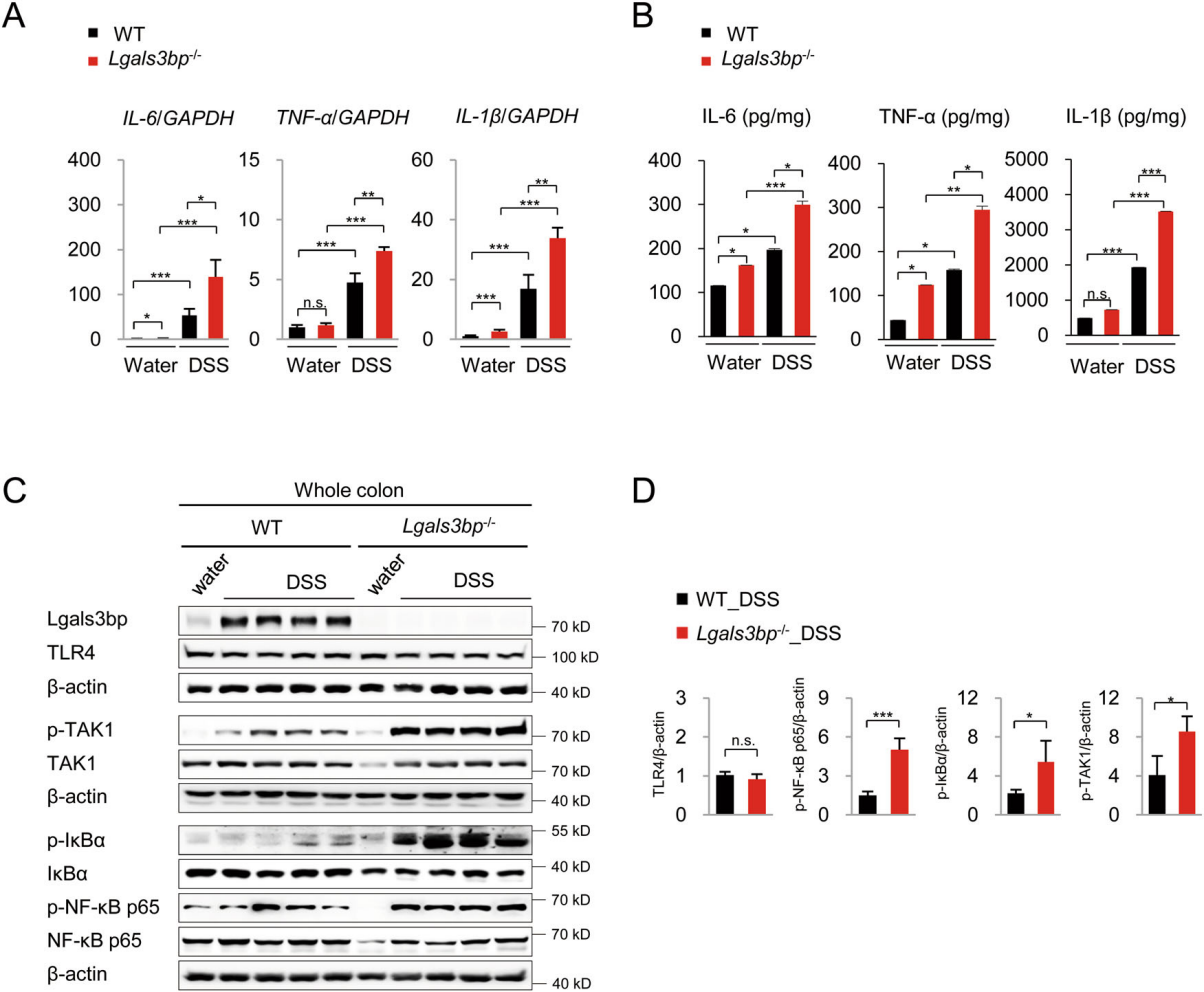
High inflammatory response in the colon during colitis due to Lgals3bp deficiency. WT (*n* = 7) and *Lgals3bp*^−/−^ (*n* = 7) mice were fed with 2.5% DSS for 7 days and sacrificed on day 7. **A** RNA was isolated from colons, and the expression of the indicated genes was analyzed by RT-qPCR. **B** The production of IL-6, TNF-α, and IL-1β in the colon homogenate was tested by ELISA. **C** Protein extracts of the whole colon from WT and *Lgals3bp*^−/−^ mice were analyzed for the activation of TAK1, NF-κB, and IκB by western blotting. **D** Densitometric analysis of band intensities of western blots are shown in (**C**). Data are presented as means ± SEM values. *p < 0.05; **p < 0.01; ***p < 0.001; ****p < 0.0001.

DSS-induced colitis development is accompanied by the increased production of pro-inflammatory factors^26–28^. To understand the role of Lgals3bp in inflammatory responses during colitis, we measured the expression of pro-inflammatory cytokines, such as IL-6, TNF-α, and IL-β using RT-qPCR. The expression of pro-inflammatory cytokines was significantly higher in the colons of *Lgals3bp*^−/−^ mice than in the colons of WT mice, and these results were consistent with those for colitis severity (Fig. 3A); this was confirmed by ELISA (Fig. 3B). The increased production of pro-inflammatory cytokines might exacerbate inflammatory responses and severity of colitis in *Lgals3bp*^−/−^ mice. As Lgals3bp negatively regulates TAK1-NF-κB signaling, which is crucial for pro-inflammatory cytokine expression, we analyzed this pathway in the colons of WT and *Lgals3bp*^−/−^ mice by western blotting. TAK1 is activated by phosphorylation of Ser and Thr residues^29^; in mice, TAK1 phosphorylation at Ser439 ensures complete activation and response to stimuli, such as LPS, TNF-α, and IL-β^30^. As shown in Fig. 3C, D, TAK1 phosphorylation at Ser439 as well as IκBα and NF-κB p65 phosphorylation were significantly increased in the colons of *Lgals3bp*^−/−^ mice, as compared to those in WT mice, indicating the hyper activation of TAK1-NF-κB signaling, associated with an enhanced inflammatory response in *Lgals3bp*^−/−^ mice.

### Lgals3bp negatively regulates the TAK1-NF-κB-cytokine axis in epithelial cells

**Fig. 4:**
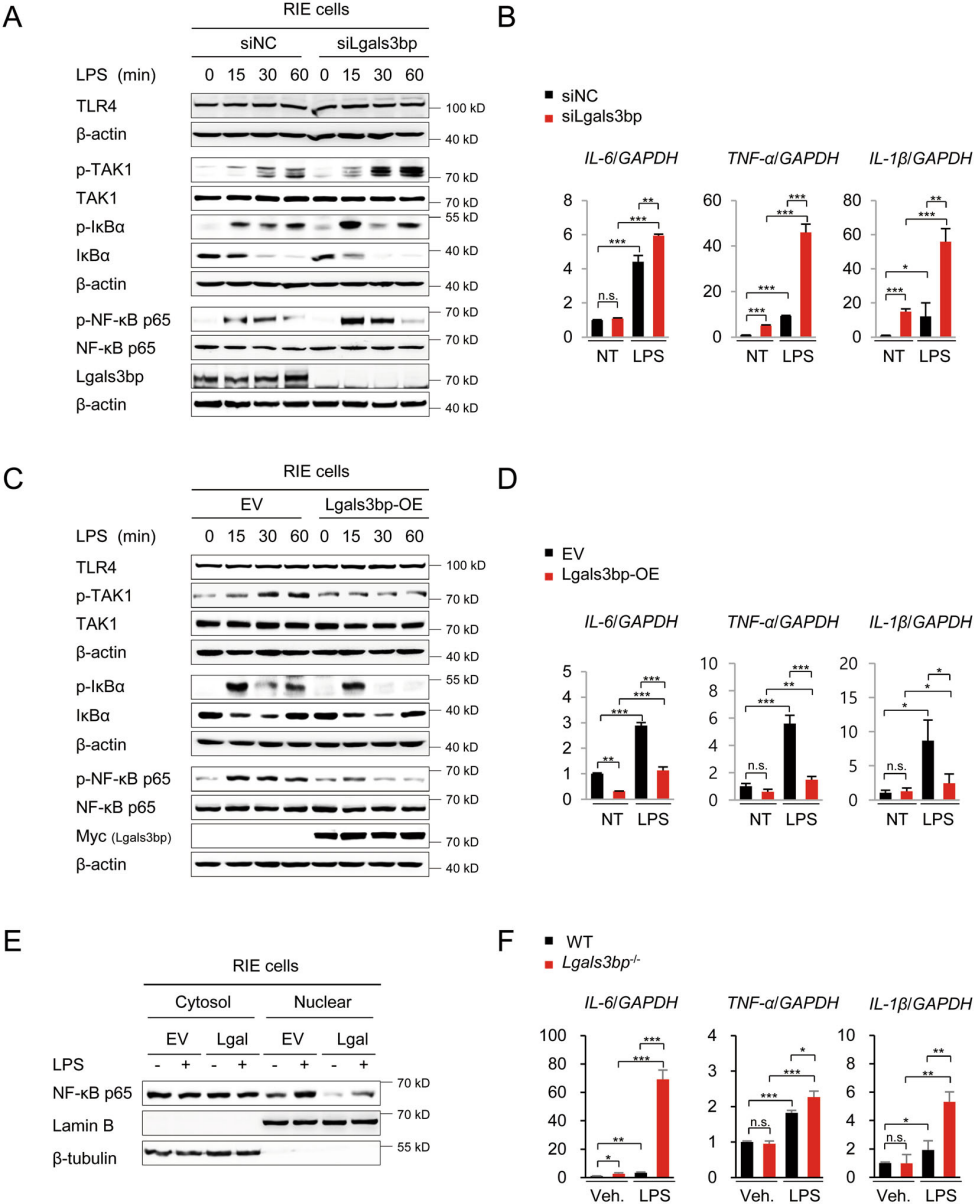
Lgals3bp-induced downregulation of NF-κB-inflammatory cytokine axis via TAK1 suppression in epithelial cells. **A**–**B** *Lgals3bp*-knockdown RIE cells were treated with LPS (100 ng/ml) for indicated periods. **A** Whole cell lysates were analyzed for the activation of TLR4, TAK1, IκB, and NF-κB via western blotting. **B** RNA was isolated from LPS-treated RIE cells, and the expression of the indicated genes was analyzed by RT-qPCR. **C**, **D** *Lgals3bp*-overexpressed RIE cells were treated with LPS (100 ng/ml) for indicated periods. **C** Whole cell lysates were analyzed for TLR4, TAK1, IκB, and NF-κB activation by western blotting. **D** RNA was isolated from LPS-treated RIE cells, and *IL-6, TNF-α*, and *IL-1β* gene expression levels were analyzed by RT-qPCR. **E** *Lgals3bp*-overexpressed RIE cells were treated with LPS (100 ng/ml) for 15 min. The expression level of NF-κB p65 was analyzed in the cytoplasmic and nuclear fractions. β–tubulin and lamin B were used as the loading controls for the cytosol and nucleus, respectively. **F** Primary colon epithelial cells were isolated from WT and *Lgals3bp*^−/−^ mice, and treated with LPS (100 ng/ml) for 3 h. The expression of indicated genes was analyzed by RT-qPCR. Data are presented as means ± SEM values. *p < 0.05; **p < 0.01; ***p < 0.001; ****p < 0.0001.

Next, we assessed the molecular mechanism underlying the effect of Lgals3bp on inflammation in colon epithelial cells. First, we analyzed TLR4-TAK1-NF-κB signaling in siNC- or si*Lgals3bp*-treated rat intestinal epithelial (RIE) cells by western blotting. As shown in Fig. 4A, LPS stimulation increased TAK1, NF-κB, and IkBα phosphorylation in *Lgals3bp*-knockdown cells, as compared to that in siNC-treated cells. Consistently, the expression of NF-κB-targets, such as IL-6, TNF-α, and IL-β was significantly higher in *Lgals3bp*-knockdown cells (Fig. 4B). We investigated whether Lgals3bp overexpression could effectively suppress TLR4-TAK1-NF-κB pathway activation in RIE cells. As expected, Lgals3bp overexpression inhibited LPS-stimulated TAK1, NF-κB, and IkBα phosphorylation (Fig. 4C) and IL-6, TNF-α, and IL-β expression (Fig. 4D). Lgals3bp-mediated NF-κB suppression was further confirmed by the decreased nuclear translocation of NF-κB p65 (Fig. 4E). We also analyzed the expression of pro-inflammatory cytokines in primary colon epithelial cells from WT and *Lgals3bp*^−/−^ mice. Upon LPS stimulation, the expression of *IL-6*, *TNF-α*, and *IL-β* mRNA was significantly higher in *Lgals3bp*^−/−^ cells than that in WT cells (Fig. 4F). These results suggested that Lgals3bp negatively regulated the NF-κB inflammatory pathway via TAK1 suppression in colon epithelial cells, thus providing protection against colitis.

### *Lgals3bp*^−/−^ mice are highly susceptible to tumorigenesis in the colon

**Fig. 5:**
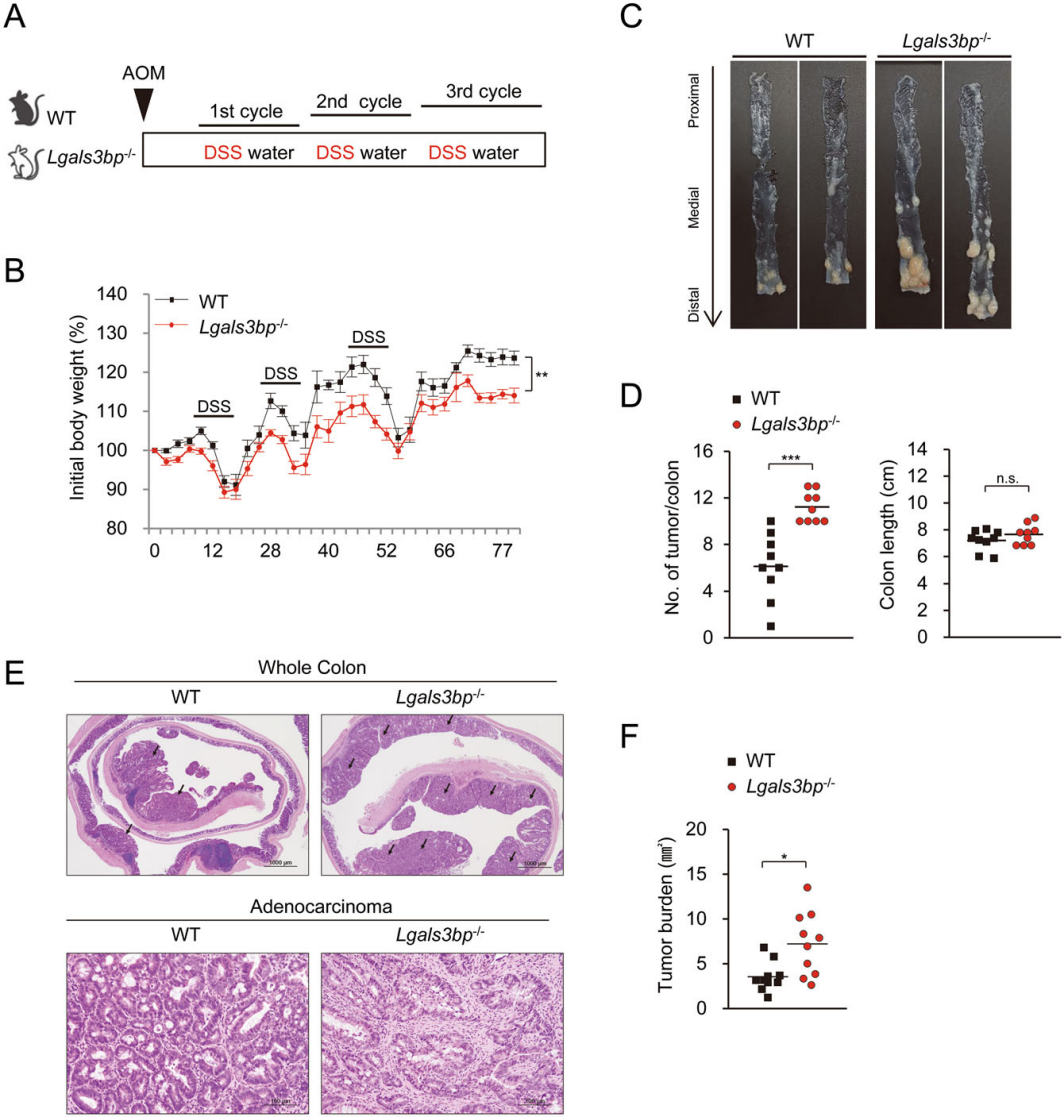
Hyper-susceptibility of *Lgals3bp*^−/−^ mice to colon tumorigenesis. **A** Schematic overview of the AOM/DSS protocol of colon tumorigenesis model. In brief, WT (*n* = 9) and *Lgals3bp*^−/−^ (*n* = 9) mice were treated intraperitoneally with AOM (10 mg/kg). After 5 days, mice were treated with 2.5% DSS in drinking water for 1 week. Thereafter, mice were fed with regular water for 2 weeks and subjected to two more DSS administration cycles. Mice were sacrificed at day 95 following AOM injection. **B** Body weight was monitored during AOM/DSS-induced tumorigenesis. **C** Representative image of tumor-bearing colons from WT and *Lgals3bp*^−/−^ mice. **D** The tumor number per colon was counted and colon length was measured. **E** Upper: Representative picture of H&E stained tumor-bearing colons. Arrows indicate tumor lesions. Lower: Representative adenocarcinoma in the colon **F** Tumor burden was measured based on histological analysis. The solid line within the graph represents the mean value. **p < 0.01.

Inflammation plays a decisive role in tumorigenesis in the colon. As shown above, Lgals3bp deficiency enhanced colon inflammation. We examined whether it also promoted inflammation-associated tumorigenesis. We induced colon tumorigenesis in WT and *Lgals3bp*^−/−^ mice with AOM/DSS (Fig. 5A). In the AOM/DSS model, mice were subjected to repeated cycles of DSS administration, which resulted in chronic inflammation and promoted AOM-induced neoplastic growth of epithelium^26^. Colitis analysis was performed by monitoring the body weight; it was significantly reduced in *Lgals3bp*^−/−^ mice, as compared to WT mice (Fig. 5B). Mice were sacrificed 95 days after AOM administration, to analyze colonic tumor incidence and burden. The number of tumors was significantly greater in *Lgals3bp*^−/−^ mice than that in WT mice, whereas there was no significant difference in colon length (Fig. 5C, D). Tumor burden was also significantly increased in *Lgals3bp*^−/−^ mice than that in WT mice (Fig. 5E, F). Together, these results suggested that Lgals3bp deficiency exacerbated tumorigenesis in the colon.

### Lgals3bp attenuates TAK1-NF-κB pathway activation in the colon during tumorigenesis

**Fig. 6:**
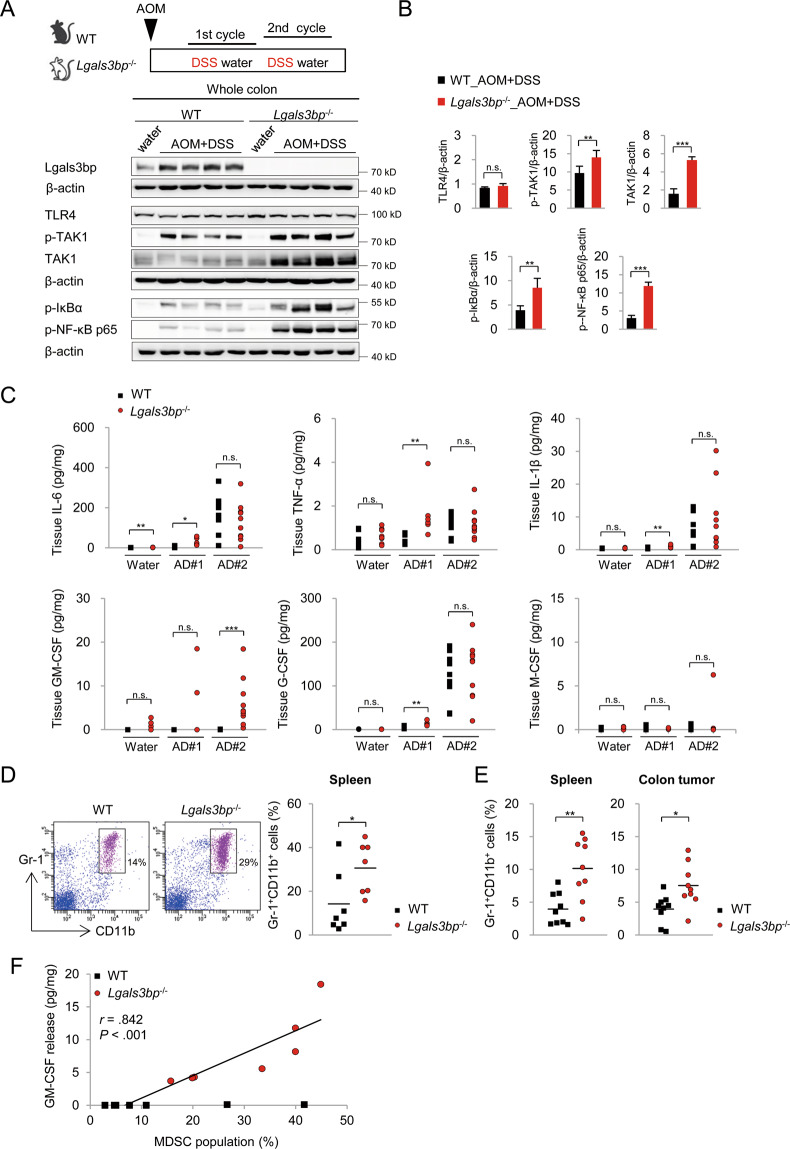
Lgals3bp deficiency-induced exacerbation of colon tumorigenesis via GM-CSF-mediated MDSC accumulation. **A** WT and *Lgals3bp*^−/−^ mice were treated with AOM (10 mg/kg). After 5 days of AOM administration, WT (*n* = 9) and *Lgals3bp*^−/−^ (*n* = 9) mice were subjected to two DSS cycles. Mice were sacrificed at day 50 following AOM injection. WT (*n* = 6) and *Lgals3bp*^−/−^ (*n* = 6) mice were subjected to one DSS cycle. Mice were sacrificed at day 12 following AOM injection. WT (*n* = 9) or *Lgals3bp*^−/−^ (*n* = 9) mice provided with water served as controls. Protein extracts of colons from WT and *Lgals3bp*^−/−^ mice were analyzed for TAK1, NF-κB, and IκB activation via western blotting. **B** Densitometric analysis of band intensity of western blots is shown in (**A**). **C** Production levels of cytokines and chemokines in colons were determined by the Luminex assay. Water, AD#1, and AD#2 indicate water feeding group, AOM with one DSS cycle group, and AOM with two DSS cycles group. **D** The total spleen cells were isolated and the MDSC population (Gr-1^+^CD11b^+^) was examined by flow cytometry. **E** AOM/DSS model mice were sacrificed at day 95 following AOM injection. The total cells of the spleen and colon tumor were isolated, and the population of MDSCs (Gr-1^+^CD11b^+^) was examined by flow cytometry. **F** The correlation between MDSC and GM-CSF abundance in the colon from tumor-bearing mice. The solid line within the graph represents the mean value. Data are presented as means ± SEM values. *p < 0.05; **p < 0.01; ***p < 0.001.

Next, we investigated whether Lgals3bp inhibited chronic inflammatory responses in the colon during tumorigenesis in a manner similar to that in acute colitis. We analyzed TAK1-NF-κB activation in colon tissues after the second cycle of DSS administration, which reflects chronic inflammation. In agreement with above-mentioned observations, TAK1, IkBα, and NF-κB p65 phosphorylation was significantly increased in *Lgals3bp*^−/−^ mice, as compared to that in WT mice (Fig. 6A, B). Interestingly, total TAK1 levels were also significantly increased in the colon of *Lgals3bp*^−/−^ mice during tumorigenesis. These data supported the observation that Lgals3bp downregulated TAK1-NF-κB signaling during colonic tumorigenesis.

### Increased GM-CSF production in the colons of *Lgals3bp*^−/−^ mice promotes tumorigenesis via MDSC expansion and infiltration

Cytokines and chemokines act as molecular switches for tumorigenesis, by reprogramming the TME^31^. To investigate critical mediators of colon tumorigenesis in *Lgals3bp*^−/−^ mice, we verified cytokine production in mouse colons after the second cycle of DSS administration using the Luminex cytokine quantification assay. Surprisingly, even though IL-6, TNF-α, and IL-β production tended to increase in *Lgals3bp*^−/−^ mice, the difference observed with WT mice was not statistically significant. Simultaneously, the production of GM-CSF, which is normally low and tightly regulated, was significantly increased in the colons of *Lgals3bp*^−/−^ mice, whereas that of the related macrophage CSF and granulocyte CSF was not increased (Fig. 6C). We further confirmed the negative regulation of GM-CSF by Lgals3bp in chronically inflamed colons and primary colon epithelial cells by RT-qPCR (Fig. S2a, b). These data supported the idea that Lgals3bp downregulated GM-CSF production in chronically inflamed colons during tumorigenesis.

**Fig. 7:**
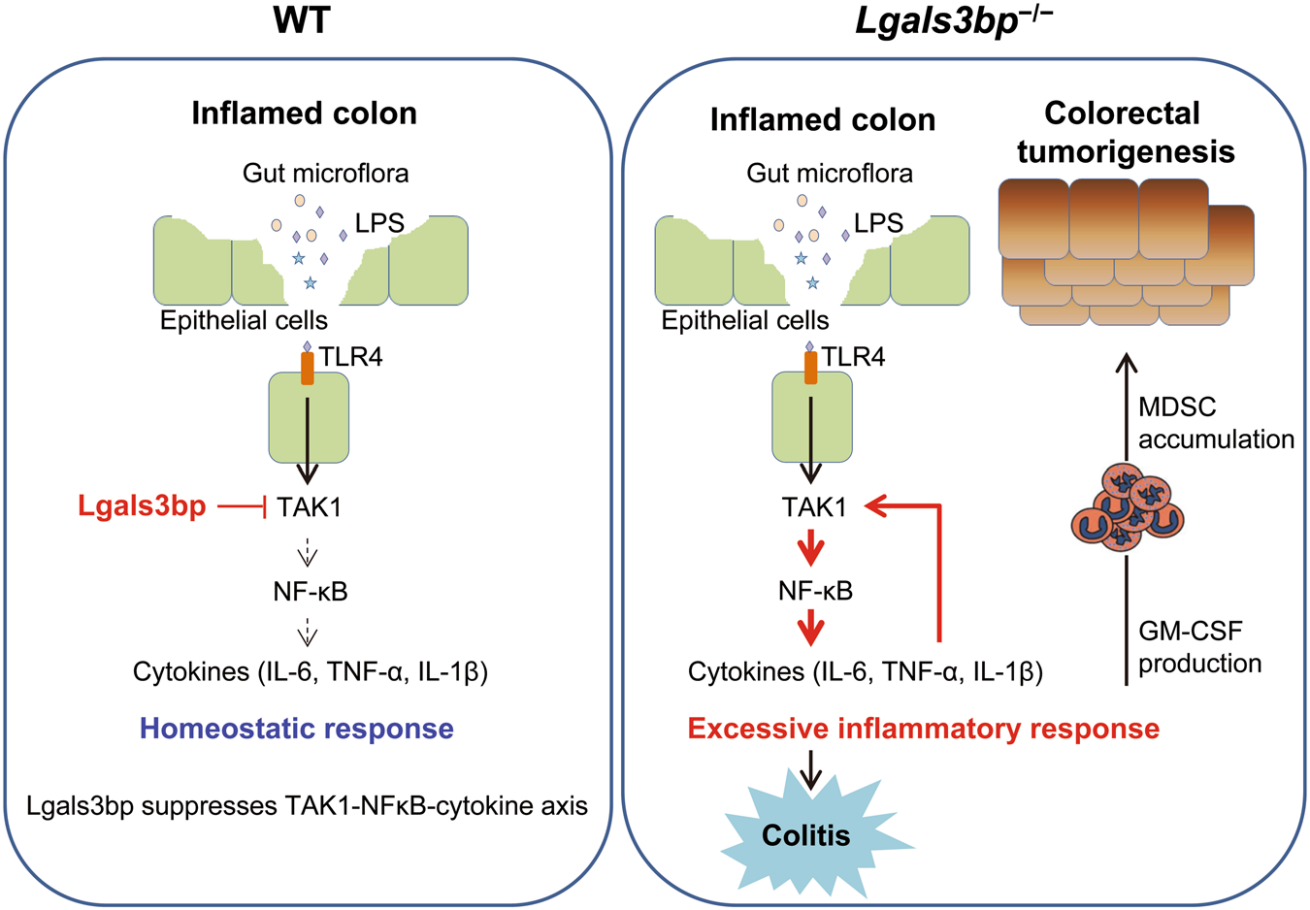
Schematic of the mechanism showing Lgals3bp-mediated regulation of colon inflammation and tumorigenesis. (1) A colonic inflammatory microenvironment is mediated by the TAK1-NF-κB-cytokine axis. Lgals3bp negatively regulates inflammatory responses via the downregulation of the TAK1-NF-κB-cytokine axis in colon epithelial cells. Therefore, Lgals3bp regulates colon homeostasis. (2) *Lgals3bp*^−/−^ mice exhibit severe colitis because of excessive pro-inflammatory cytokine production. Furthermore, *Lgals3bp*^−/−^ mice exhibit severe colon tumorigenesis via GM-CSF-mediated MDSC accumulation.

As GM-CSF is involved in MDSC accumulation during tumorigenesis in the colon, we analyzed the MDSC population in the mouse spleens after the second cycle of DSS administration. Consistent with the high level of GM-CSF, the MDSC population was significantly increased in *Lgals3bp*^−/−^ mice, as compared to that in WT mice (Fig. 6D). To verify tumor infiltration of MDSCs, we analyzed the MDSC population in colon cancer tissues and spleens at the endpoint of AOM/DSS model. As shown in Fig. 6E, the ratios of tumor-infiltrated and splenic MDSCs were significantly increased in *Lgals3bp*^−/−^ mice. As expected, the MDSC population was positively correlated with GM-CSF production (Fig. 6F). Overall, these findings suggested that increased GM-CSF production in the colon of *Lgals3bp*^−/−^ mouse promoted tumorigenesis via MDSC accumulation. We, therefore, propose that the elevated production of GM-CSF in *Lgals3bp*^−/−^ mice due to the increased activation of TAK1-NF-κB signaling leads to MDSC accumulation and stimulation of tumorigenesis in the colon (Fig. 7).

## Discussion

We have shown that Lgals3bp negatively regulates colonic inflammation and tumorigenesis by suppressing TAK1-NF-κB signaling in the colon epithelium. This suggests that Lgals3bp exhibits protective activities against inflammation and tumorigenesis in the colon.

The involvement of luminal flora and TLR signaling is a unique aspect of colonic inflammation and tumorigenesis^1,32^. LPS is abundantly released by the colonic bacteria and interacts with TLR4, which activates the TAK1-NF-κB-cytokine axis, and ultimately promotes inflammation and tumorigenesis in the colon. Lgals3bp negatively regulated these signals through TAK1 suppression. NF-κB reportedly mediated TNF-α-induced *Lgals3bp* mRNA expression in breast cancer cells^33^. In agreement with this result, Lgals3bp was upregulated in the NF-κB-activated inflamed colon. Investigating the phenotypes of Lgals3bp knockout is crucial for understanding its function in the colon. *Lgals3bp*^−/−^ mice were highly susceptible to inflammation and tumorigenesis in the colon. This study furthers our understanding of the negative regulation of inflammation in the colon, where Lgals3bp inhibits TAK1-NF-κB-cytokine signaling to prevent excessive inflammation and tumorigenesis.

Epithelial cells, the major cell type in the colon, act as gatekeepers of gut immune homeostasis and provide a barrier against luminal bacterial flora. Our results show that levels of Lgals3bp, expressed in colon epithelial cells, are further increased by inflammation. Macrophages provide robust inflammatory responses via TLR activation in the lamina propria of the gut^26,34^. Given that *Lgals3bp* is also expressed in macrophages^15^, we conclude that Lgals3bp suppresses inflammatory responses in both epithelial cells and gut macrophages.

TAK1 has emerged as a therapeutic target for inflammatory disorders, fibrosis, and cancer^9^. TAK1 inhibition suppresses inflammation and fibrosis in the colon, kidney, and lung^30,35,36^ and inhibits the in vivo growth of KRAS-mutated colon cancer cells and pro-survival signaling in breast cancer cells^37,38^. Furthermore, TAK1 inhibitors reduce chemoresistance in pancreatic, ovarian, and esophageal cancer cells^39–41^. As TAK1 is a key kinase linking exogenous stimuli and cellular responses, cancer cells utilize it to adapt to the TME. TAK1 targeting is effective in inhibiting breast cancer lung metastasis, via the suppression of IL-1 secretion^42^. With the recent interest in TAK1 as a target for inflammatory diseases and cancer, selective and orally active inhibitors, including LYTAK1 and oxindole derivatives, are currently under development^9^. Cellular Lgals3bp directly interacts with TAK1, and reduces protein stability and the binding affinity between TAK1 and its adaptor proteins^15^. Other studies suggested that Lgals3bp exhibited antiviral activity, and reported about the interaction between Lgals3bp and TAK1^14^. Here, we revealed that Lgals3bp is an endogenous negative regulator of TAK1 in the colon and suggest that Lgals3bp induction or use of the Lgals3bp-based peptide might be a potential therapeutic approach for IBD and colon cancer. Further studies are necessary to map the region of Lgals3bp responsible for TAK1 interaction and suppression.

In the TME, MDSCs contribute to tumorigenesis and cancer immune evasion by suppressing the anti-tumor functions of T cells^43^. Therefore, it is important to identify factors influencing MDSC differentiation, recruitment, and activation. GM-CSF has been linked to MDSC generation in tumorigenesis, and elevated GM-CSF production has been observed in the mucosa of IBD patients and in a mouse model of colitis^23,44^. Colon epithelial cells are a major cellular source of GM-CSF production in the AOM/DSS model. GM-CSF is significantly increased in the chronic inflamed colon and its blockade suppresses MDSC activation and cancer development^23^. Here, we found that Lgals3bp deficiency promoted colonic tumorigenesis through an increase in GM-CSF and accumulation of MDSCs. Because GM-CSF also promoted tumor-associated macrophage (TAM) activation^45^, it should be investigated whether LGALS3BP suppresses pro-tumor cells, including TAMs, through the downregulation of GM-CSF and other cytokines in the TME.

Although LGALS3BP was originally identified in cancer cells, its role in tumorigenesis remains unclear. LGALS3BP reportedly has both negative and positive influences on various cancer prognoses. For example, though high serum LGALS3BP levels are associated with poor clinical outcomes in patients with breast, hepatocellular, and non-small cell lung carcinoma^16,46,47^, high LGALS3BP expression in tumor tissues is associated with favorable outcomes in patients with Ewing’s sarcoma and CRC^21,22^. Thus, our results suggest that Lgals3bp suppresses colonic tumorigenesis via TAK1 downregulation. Based on the various functions of TAK1, LGALS3BP might inhibit tumorigenesis and cancer progression by suppressing tumor cell proliferation, metastasis, and anti-cancer drug resistance. Therefore, further studies are required to investigate the role of LGALS3BP in various aspects of cancer.

In conclusion, this study demonstrates that Lgals3bp is a physiological regulator of inflammation and tumorigenesis in the colon. We provided molecular evidence that Lgals3bp-mediated protection against colonic inflammation and tumorigenesis reflects its unique function of inhibiting TAK1-NF-κB signaling and that Lgals3bp deficiency advances colon tumorigenesis by increasing GM-CSF production and MDSC accumulation. Therefore, the Lgals3bp-mediated negative regulation of TAK1 might be a key target for colon cancer immunotherapy.

## Material and methods

### Reagents and antibodies

Lipopolysaccharide (LPS) from *Escherichia coli* 055:B5 (L2880) and azoxymethane (AOM; A5486) were purchased from Sigma-Aldrich (St. Louis, MO, USA). Dextran sodium sulfate (DSS; 160110) was purchased from MP Biomedicals (Solon, OH, USA). Other chemical reagents were obtained from Sigma-Aldrich.

### Cell culture and transfection

Rat intestinal epithelial (RIE) cells (generously gifted by Prof. Young Eun Joo) were cultured in Dulbecco’s modified Eagle’s medium (Invitrogen, Carlsbad, CA, USA) supplemented with 10% fetal bovine serum (FBS) and 1% penicillin-streptomycin (Invitrogen). Lgals3bp and negative control siRNAs were purchased from Bioneer Corp. (Daejeon, Republic of Korea). siRNA and DNA were transfected using Lipofectamine RNAiMAX (Invitrogen; 13778150) and Lipofectamine 3000 (Invitrogen, L3000015), respectively according to the manufacturer’s protocol. For transient transfection, RIE cells were grown to 70% confluence in a 6-well plate. Lipofectamine 3000 (3.75 µL) was diluted and mixed with 150 µL of Opti-MEM (Invitrogen; 11058021). Plasmid DNA (2.5 µL) was diluted in 150 µL of Opti-MEM and then 5 µL of P3000 was added to it. The two mixtures were then combined and incubated for 5 min. The transfection mixture was added to RIE cells. For siRNA-mediated knockdown of Lgals3bp, RIE cells were transfected with 30 nM of either the control or Lgals3bp siRNA using RNAiMAX for 48 h. Briefly, siRNA and RNAiMAX were diluted in Opti-MEM. The two mixtures were then combined and incubated for 5 min. The RIE cells were treated with the transfection mixture. The sequences of siRNAs targeting Lgals3bp were as follows: sense: 5′-GAGACUUCCUCAGGUACUUtt-3′; antisense: 5′-AAGUACCUGAGGAAGUCUCtt-3′.

### Mice and colitis and tumorigenesis models

*Lgals3bp*^−/−^ C57BL/6N mice were generated using the CRISPR-Cas9 genome editing system (Macrogen, Inc., Seoul, Korea) as described previously^15^. All experimental mice were housed in a specific, pathogen-free facility. All animal protocols were approved by The Chonnam National University Medical School Research Institutional Animal Care and Use Committee (IACUC; CNU-IACUC-H-2017-50). Experiments were conducted in accordance with the IACUC guidelines. The colitis model was established by applying 2.5% DSS to the drinking water for 7 days. Colon tumorigenesis was induced using the AOM/DSS model, as described previously^26^. C57BL/6N mice (6–8 weeks old, male) were used in all experiment.

### Colitis index scoring

The criteria for assessing colitis severity included body weight loss, stool consistency, and rectal bleeding. Body weight loss scores (0–4) were as follows: 0, none; 1, 1–5% loss; 2, 6–10% loss; 3, 11–20% loss; 4, >20% loss. Stool consistency was scored as follows: 0, normal; 2, soft (loose); 3, diarrhea. Rectal bleeding was scored as follows: 0, none; 1, occult bleeding; 3, red; 4, dark red; 5, gross bleeding.

### TUNEL and histopathological analysis

Colons were fixed in 4% paraformaldehyde and embedded in paraffin. TUNEL assays were performed using the Dead-End fluorometric TUNEL system (Promega, Madison, WC, USA; G3250), according to the manufacturer’s protocol. Colon sections were stained with hematoxylin and eosin (H&E). Histopathological scoring was performed in a blinded fashion by an experienced pathologist [Lee KH]. Histological scoring was based on four parameters. The severity of lymphoid follicles and hyperplasia was scored as follows: 0, normal; 1, mild; 2, moderate; 3, severe. Inflammation severity was scored as follows: 0, rare inflammatory cells in lamina propria; 1, increased numbers of leukocytes in lamina propria; 2, confluence of inflammatory cells extending into the submucosa; 3, transmural extension of the inflammatory infiltrate. Areas of epithelial loss, indicative of ulcers/erosion and changes in the crypt architecture, including flattening atypia and glandular complexity, were measured within colon sections.

### Isolation of colonic epithelial cells and lamina propria

To isolate colonic epithelial cells, colons were sectioned into small pieces and digested in Digestion Buffer 1 (RPMI 1640 containing 5% FBS, 5 mM EDTA, and 1 mM dithiothreitol) at 37 °C. The remaining tissues were further digested in Digestion Buffer 2 (RPMI 1640 containing 5% FBS, collagenase, and DNase1) at 37 °C for 50 min. The homogenate was filtered with a 70 µm Nylon cell strainer (BD Biosciences, Bedford, MA, USA), washed with Washing Buffer 1 (ice-cold PBS containing 0.2% EDTA), and re-suspended in Washing Buffer 2 (ice-cold PBS containing 3% FBS and 0.2% EDTA).

### Flow cytometry

Cells isolated from tumors, spleens, and red blood cells were removed with lysis buffer (Sigma; R7757). Cell suspensions in FACS buffer (1% BSA/PBS) were blocked for 15 min with CD16/32 antibodies (eBioscience, INC. San Diego, CA). Cells were stained for 30 min at 4 °C with fluorescently-conjugated CD11b and Gr-1 antibodies. Samples were analyzed using FACScanto (BD PharMingene) and FlowJo Software (Treestar, Ashland, OR).

### Nuclear and cytoplasmic fractionation

Nuclear and cytoplasmic fractions were prepared using the NE-PER Nuclear and Cytoplasmic Extraction Reagent (Thermo Fisher Scientific, Waltham, MA, USA; 78835), according to the manufacturer’s instructions.

### Western blot analysis

Whole cell lysates were prepared with a protein extraction reagent (Thermo Fisher Scientific; 78501). Equal protein amounts were boiled, separated by SDS-PAGE, and transferred onto a PVDF membrane. Membranes were blocked using SuperBlock^TM^ T20 blocking buffer (Thermo Fisher Scientific; 37515) and incubated overnight at 4 °C with primary antibodies (Supplementary Table 1). Horseradish peroxidase-conjugated secondary antibodies were used to probe the membranes for 1 h at 25 °C, and the membranes were visualized using a low-light imaging system (LAS-4000 mini; FUJIFILM Medical Systems). Band intensities were quantified using the Multi-Gauge 3.0 software.

### PCR and real-time quantitative (RT-q) PCR

Total RNA was isolated using the Hybrid-R reagent (305-101; GeneAll Biotechnology, Seoul, Korea). Equal amounts of cDNA were synthesized with 500 ng of sample RNA, using the GoScript Reverse Transcription Mix (Promega; A2791). PCR was performed using GoTaq DNA polymerase (Promega; M300F) and gene-specific primer pairs. RT-qPCR was performed using Quantifast SYBR Green PCR Master Mix (Qiagen, Hilden, Germany; 204054) on a RotorGene 3000 (Qiagen) machine. Primers are listed in Supplementary Table 2.

### Cytokine analysis

Cytokine levels in tissue homogenates were measured using commercial enzyme-linked immunosorbent assay (ELISA) kits for mouse IL-6 (51-25632E), IL-1β (51-26662E), and TNF-α (51-26732E) (BD Biosciences, San Jose, CA, USA). Alternatively, cytokines and chemokines were quantified with the multiplex assay (EMD Millipore, MCYTOMAG-70K), according to the manufacturer’s instructions.

### Statistical analysis

Statistical analysis was performed using SPSS 12.0 (SPSS, Inc., Chicago, IL, USA). Statistical significance was assessed by Mann–Whitney *U* test. Survival analysis was performed using the Kaplan–­Meier method. Statistically significant were considered when *P* < 0.05.

## Supplementary information

Table S1.

Table S2.

Figure S1.

Figure S2.

Figure S3.

## Data Availability

The datasets used and analyzed during the current study are available from the corresponding author on reasonable request.
