# Supplementary material for: Lgals3bp suppresses colon inflammation and tumorigenesis through the downregulation of TAK1-NF-κB signaling
Source: Cell Death Discov. 2021 Apr 6;7:65. doi: 10.1038/s41420-021-00447-7 (PMC8024364; doi:10.1038/s41420-021-00447-7)
Supplement: Supplementary file 1 — Table S1. [file 41420_2021_447_MOESM1_ESM.docx]

### **Table S1. List of antibodies**

| Primary antibodies | Manufacturer | Catalog No. | Applications | Dilution |
| --- | --- | --- | --- | --- |
| TAK1 | Cell Signaling Technology | 5206 | WB | 1:1000 |
| p-IKKαβ | Cell Signaling Technology | 2697 | WB | 1:1000 |
| IkBα | Cell Signaling Technology | 4814 | WB | 1:1000 |
| p-IkBα | Cell Signaling Technology | 9246 | WB | 1:1000 |
| p-NF-kB p65 | Cell Signaling Technology | 3033 | WB | 1:1000 |
| NF-kB p65 | Cell Signaling Technology | 6956 | WB | 1:1000 |
| Lamin B | Cell Signaling Technology | 13435 | WB | 1:1000 |
| p-TAK1 | Abcam | ab109404 | WB | 1:1000 |
| p-TAK1 | Millipore | 06-1425 | WB | 1:1000 |
| β-actin | Abcam | ab6276 | WB | 1:1000 |
| β-tubulin | Abcam | ab52901 | WB | 1:1000 |
| TLR4 | Santa Cruz | sc-293072 | WB | 1:1000 |
| Lgals3bp | IBL | 28125 | WB | 1:1000 |
| CD11b-APC | BD Pharmingen | 553312 | Flow cytometry | - |
| Gr-1-PerCP | BD Pharmingen | 552093 | Flow cytometry | - |

All secondary HRP-linked antibodies for Western Blot analysis were purchased from Jackson ImmunoResearch Laboratories. All antibodies were diluted and used according manufacturer’s recommendations
