# Supplementary material for: Lgals3bp suppresses colon inflammation and tumorigenesis through the downregulation of TAK1-NF-κB signaling
Source: Cell Death Discov. 2021 Apr 6;7:65. doi: 10.1038/s41420-021-00447-7 (PMC8024364; doi:10.1038/s41420-021-00447-7)
Supplement: Supplementary file 2 — Table S2. [file 41420_2021_447_MOESM2_ESM.docx]

Table S2: List of primers

| Gene | Primers Sequences (5’-3’) F/R | Accession No. | Applications |
| --- | --- | --- | --- |
| *mIL-6* | TTCTCCACAAGCGCCTTCGGTC  CTGTGTGGGGCGGCTACATCT | NM_000610.4 | RT-qPCR |
| *mTNF-α* | TCTTCTCGAACCCCGAGTGA CCTCTGATGGCACCACCAG | NM_002046.7 | RT-qPCR |
| *mIL-1β* | CCCTGCAGCTGGAGAGTGTGGA TGTGCTCTGCTTGTGAGGTGCTG | NM_008361.4 | RT-qPCR |
| *mGM-CSF* | ATGCCTGTCACGTTGAATGAAG  GCGGGTCTGCACACATGTTA | NM_009969.4 | RT-qPCR |
| *mLgals3bp* | TGCTGGTTCCAGGGACTCAA  CCACCGGCCTCTGTAGAAGA | NM_011150.3 | RT-qPCR,  RT-PCR |
| *mLgals3bp* | TGGAGACATGCGCTTGGTT  GTGAGGACTCGGTCCCTGTA | NM_011150.3 | Knockout confirmation |
| *mGAPDH* | AGGTCGGTGTGAACGGATTTG  TGTAGACCATGTAGTTGAGGTCA | NM_008084.3 | RT-qPCR |
| *rIL-6* | TGATGGATGCTTCCAAACTG  GAGCATTGGAAGTTGGGGTA | NM_012589.2 | RT-qPCR |
| *rTNF-α* | ACTGAACTTCGGGGTGATCG  GCTTGGTGGTTTGCTACGAC | NM_012675.3 | RT-qPCR |
| *rIL-1β* | CACCTTCTTTTCCTTCATCTTTG  GTCGTTGCTTGTCTCTCCTTGTA | NM_031512.2 | RT-qPCR |
