# Supplementary figures and images for: Lgals3bp suppresses colon inflammation and tumorigenesis through the downregulation of TAK1-NF-κB signaling
Source: Cell Death Discov. 2021 Apr 6;7:65. doi: 10.1038/s41420-021-00447-7 (PMC8024364; doi:10.1038/s41420-021-00447-7)

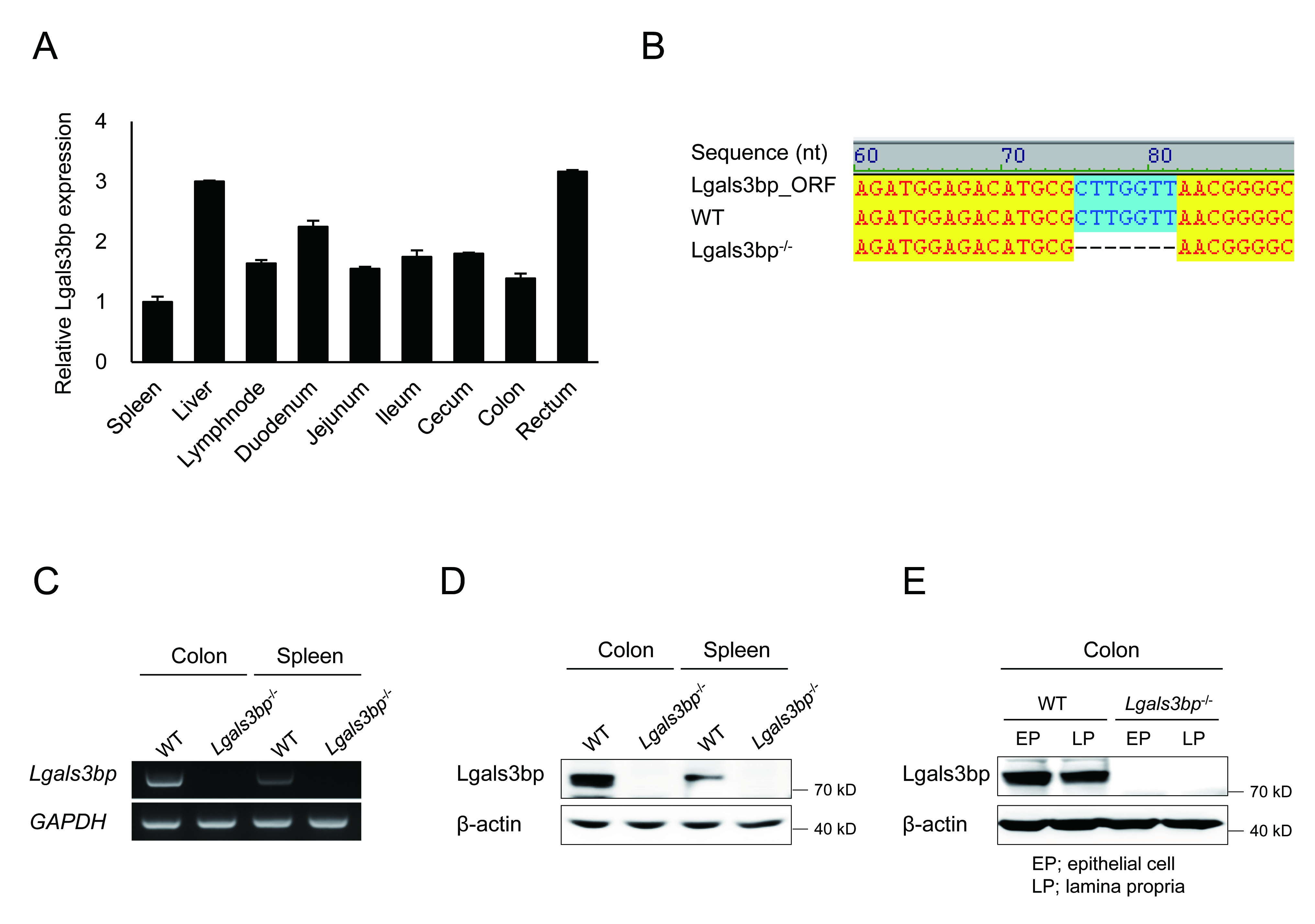

Supplement: Supplementary file 3 — Figure S1. [file 41420_2021_447_MOESM3_ESM.tif]

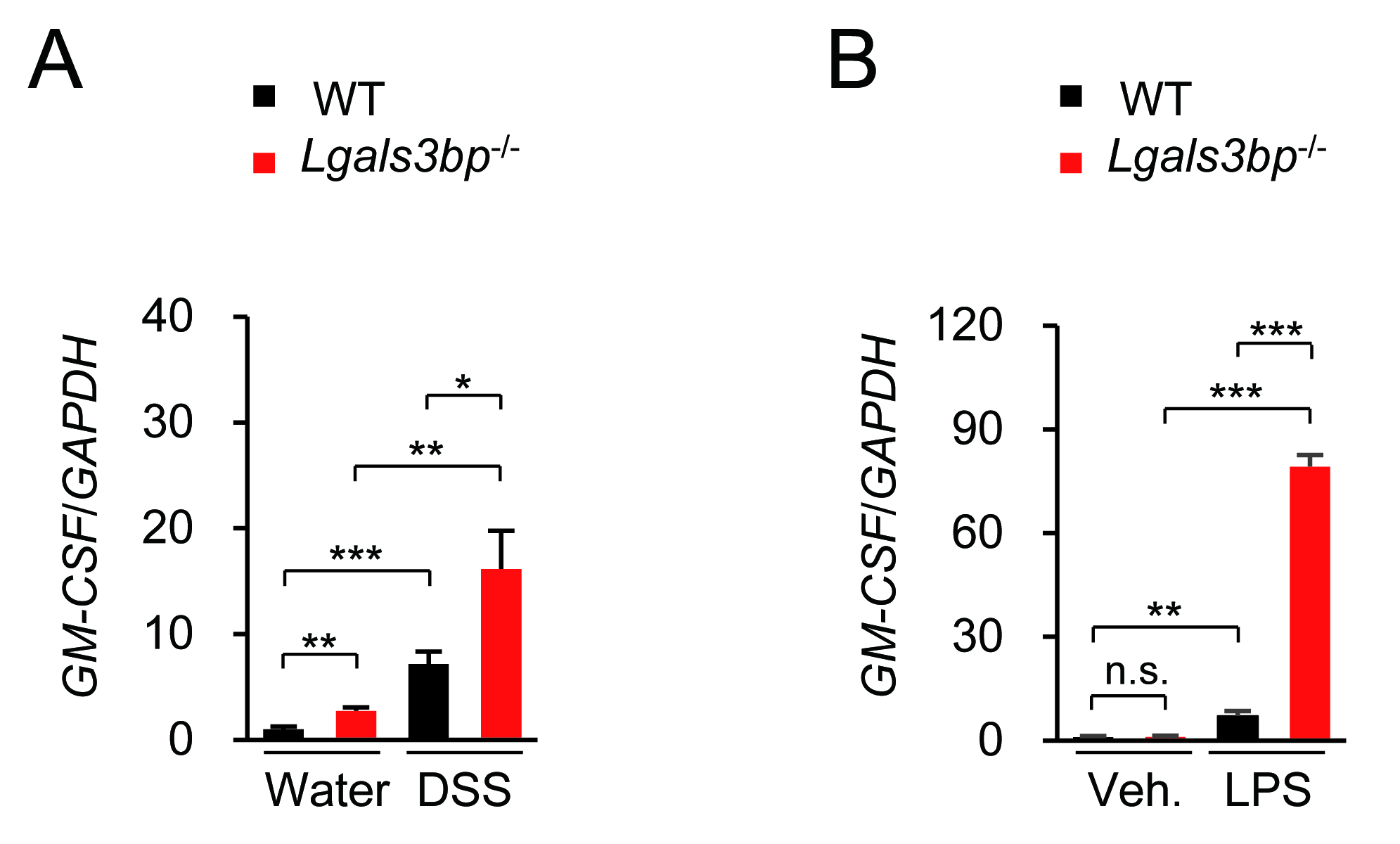

Supplement: Supplementary file 4 — Figure S2. [file 41420_2021_447_MOESM4_ESM.tif]
