# Supplementary material for: Lgals3bp suppresses colon inflammation and tumorigenesis through the downregulation of TAK1-NF-κB signaling
Source: Cell Death Discov. 2021 Apr 6;7:65. doi: 10.1038/s41420-021-00447-7 (PMC8024364; doi:10.1038/s41420-021-00447-7)

Fig 1D

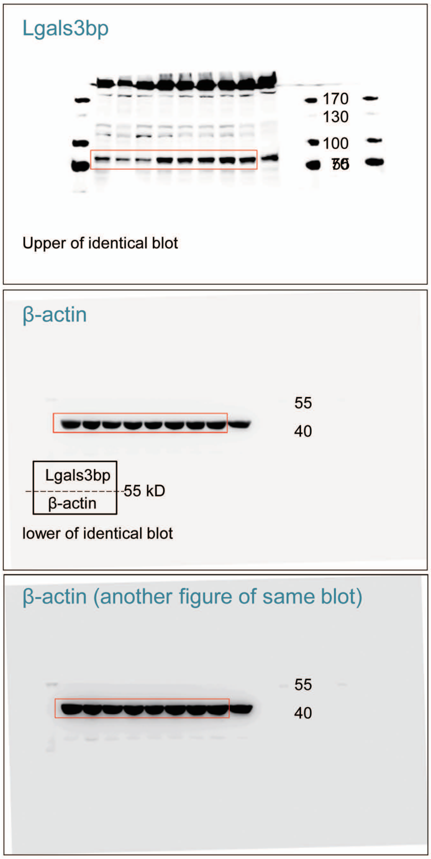

Lgals3bp
170
130
100
70
55
Upper of identical blot
β-actin
55
40
Lgals3bp
β-actin
55 kD
lower of identical blot
β-actin (another figure of same blot)
55
40

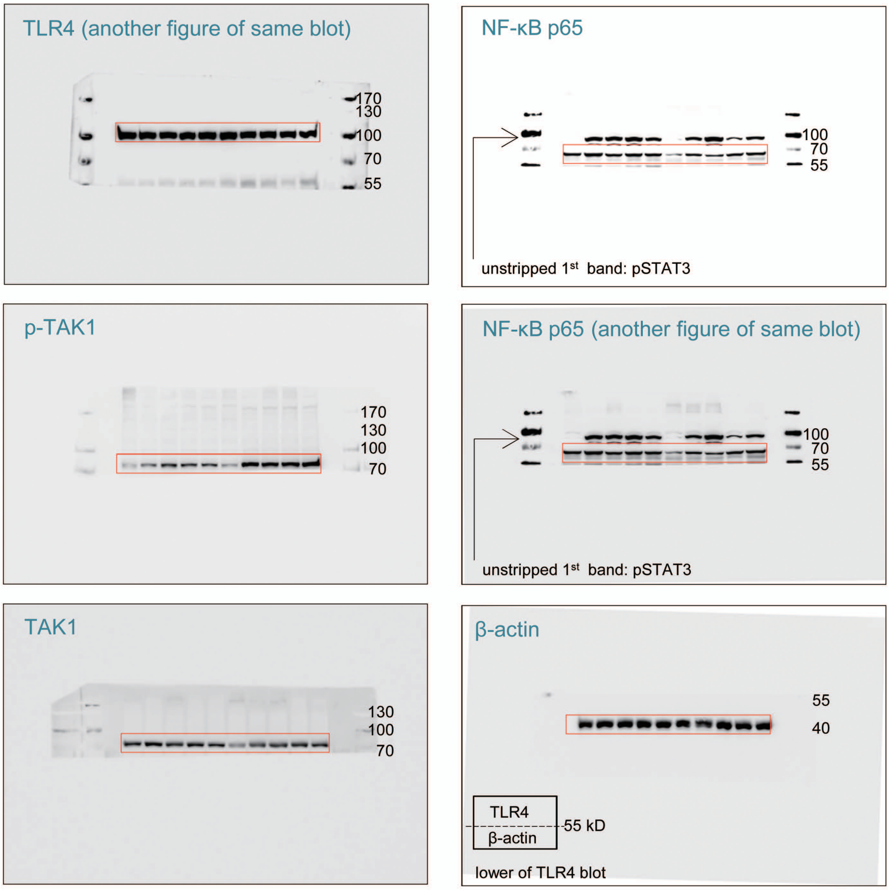

TLR4 (another figure of same blot)
170
130
100
70
55
p-TAK1
170
130
100
70
TAK1
130
100
70
NF-κB p65
100
70
55
unstripped 1st band: pSTAT3
NF-κB p65 (another figure of same blot)
100
70
55
unstripped 1st band: pSTAT3
β-actin
55
40
TLR4
β-actin
55 kD
lower of TLR4 blot

Fig 3C

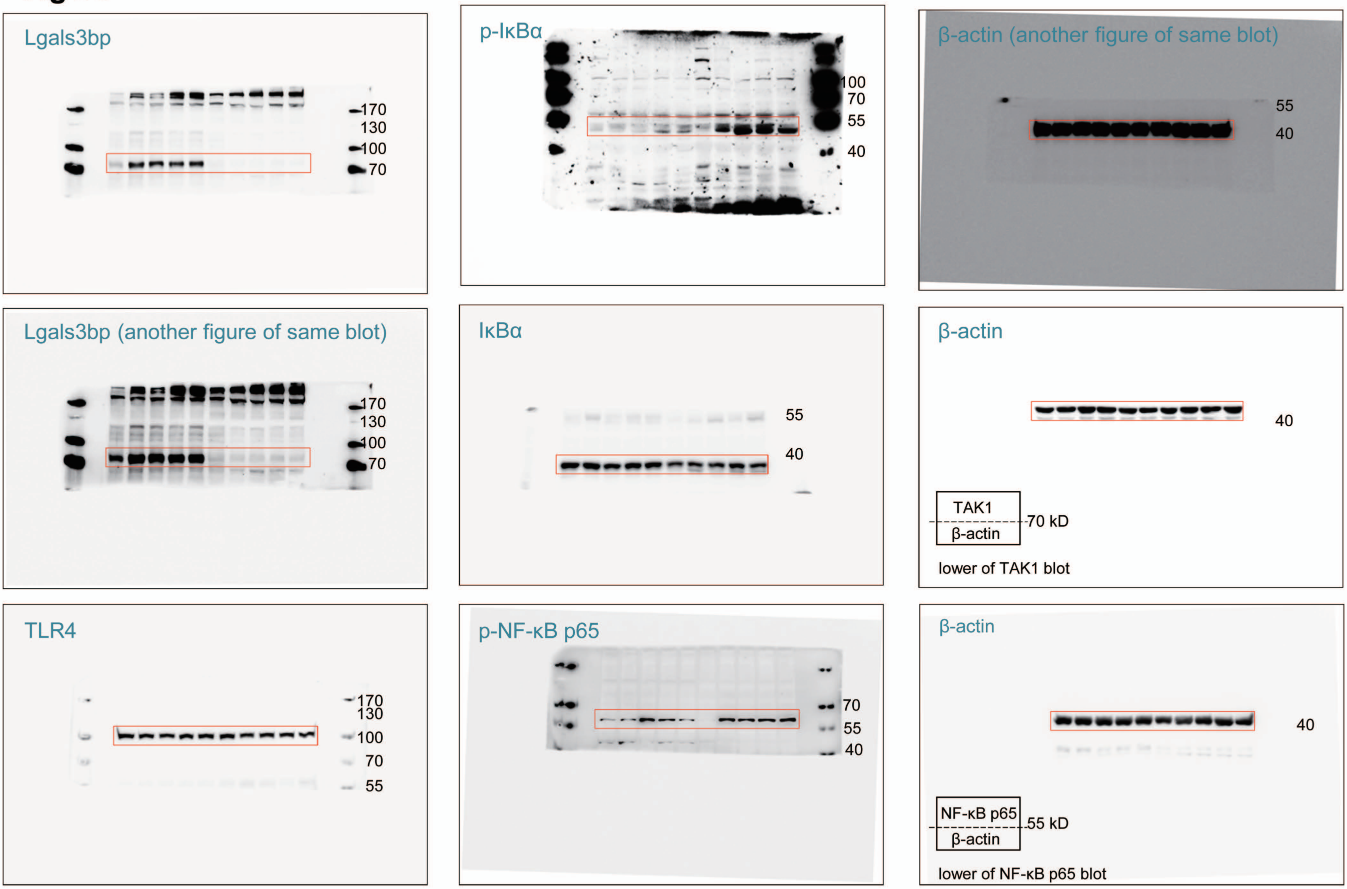

Lgals3bp
170
130
100
70
Lgals3bp (another figure of same blot)
170
130
100
70
TLR4
170
130
100
70
55
p-IκBα
100
70
55
40
IκBα
55
40
p-NF-κB p65
70
55
40
β-actin (another figure of same blot)
55
40
β-actin
40
TAK1
β-actin
70 kD
lower of TAK1 blot
β-actin
40
NF-κB p65
β-actin
55 kD
lower of NF-κB p65 blot

Fig 4A

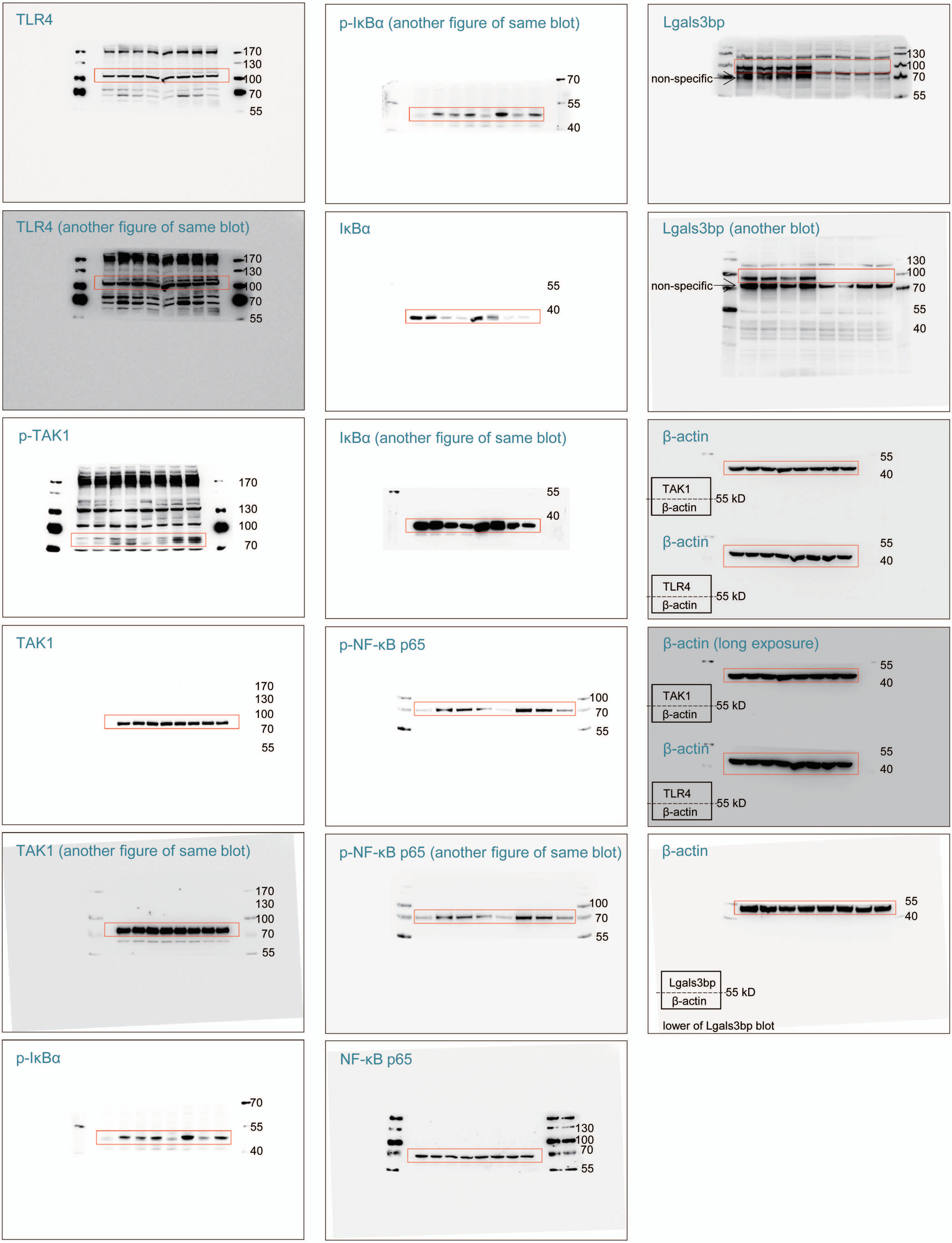

TLR4
170
130
100
70
55
p-IκBα (another figure of same blot)
70
55
40
Lgals3bp
non-specific
130
100
70
55
TLR4 (another figure of same blot)
170
130
100
70
55
IκBα
55
40
Lgals3bp (another blot)
non-specific
130
100
70
55
40
p-TAK1
170
130
100
70
IκBα (another figure of same blot)
55
40
β-actin
55
40
TAK1
β-actin
55 kD
β-actin
55
40
TLR4
β-actin
55 kD
TAK1
170
130
100
70
55
p-NF-κB p65
100
70
55
β-actin (long exposure)
55
40
TAK1
β-actin
55 kD
β-actin
55
40
TLR4
β-actin
55 kD
TAK1 (another figure of same blot)
170
130
100
70
55
p-NF-κB p65 (another figure of same blot)
100
70
55
β-actin
55
40
Lgals3bp
β-actin
55 kD
lower of Lgals3bp blot
p-IκBα
70
55
40
NF-κB p65
130
100
70
55

Fig 4C

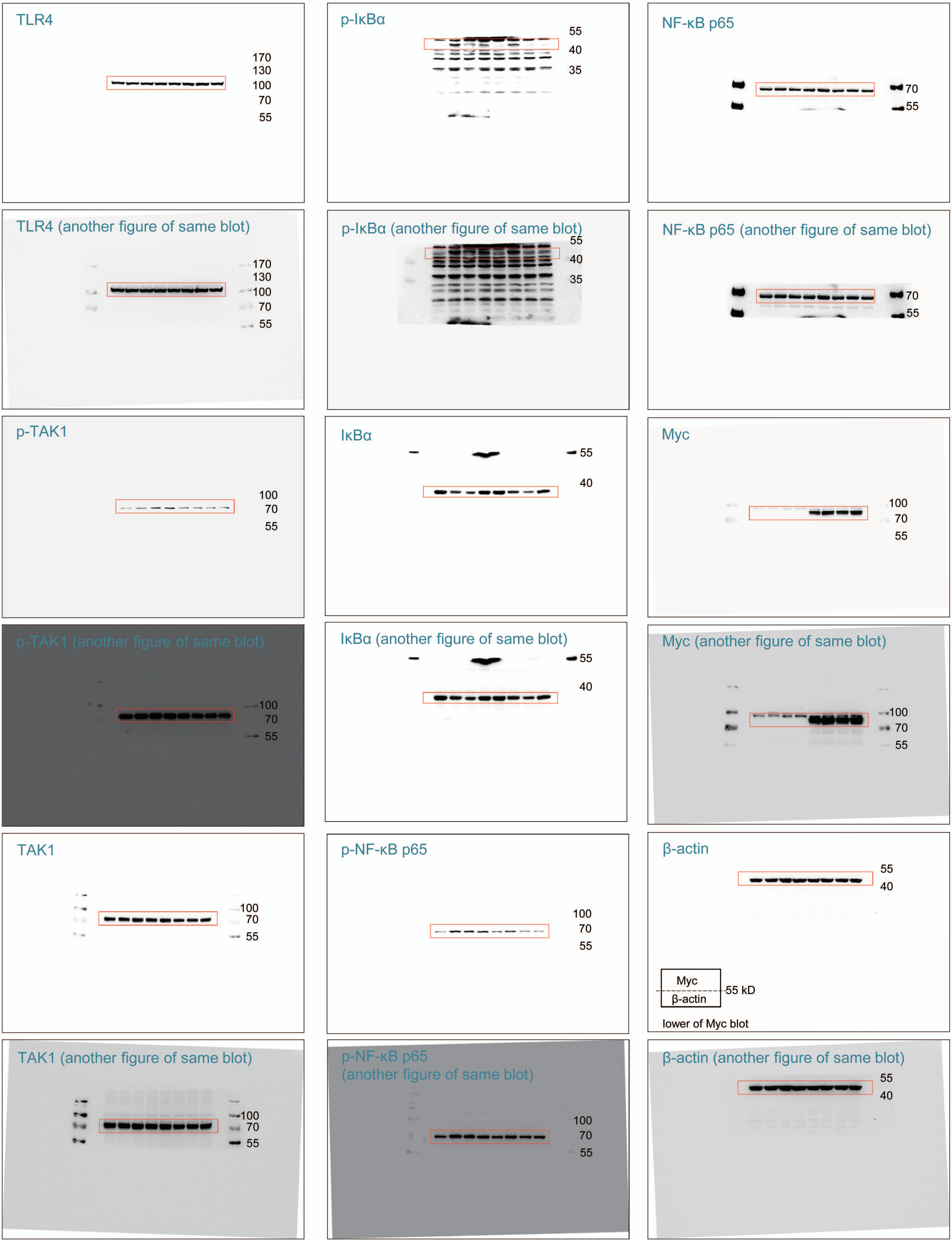
TLR4
170
130
100
70
55
p-IκBα
55
40
35
NF-κB p65
70
55
TLR4 (another figure of same blot)
170
130
100
70
55
p-IκBα (another figure of same blot)
55
40
35
NF-κB p65 (another figure of same blot)
70
55
p-TAK1
100
70
55
IκBα
55
40
Myc
100
70
55
p-TAK1 (another figure of same blot)
100
70
55
IκBα (another figure of same blot)
55
40
Myc (another figure of same blot)
100
70
55
TAK1
100
70
55
p-NF-κB p65
100
70
55
β-actin
55
40
Myc
β-actin
55 kD
lower of Myc blot
TAK1 (another figure of same blot)
100
70
55
p-NF-κB p65
(another figure of same blot)
100
70
55
β-actin (another figure of same blot)
55
40

Fig 4C (continued)

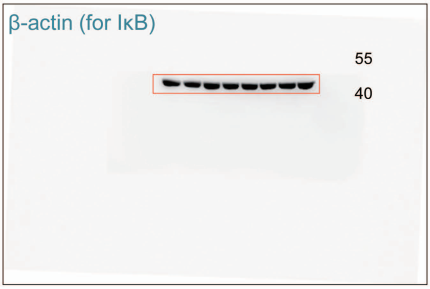

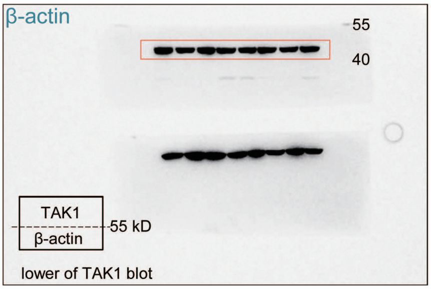

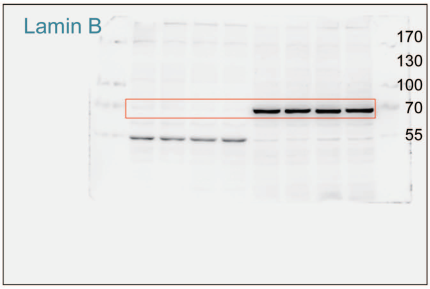

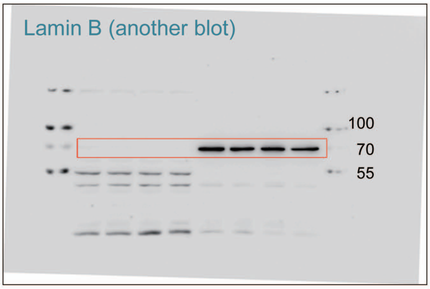

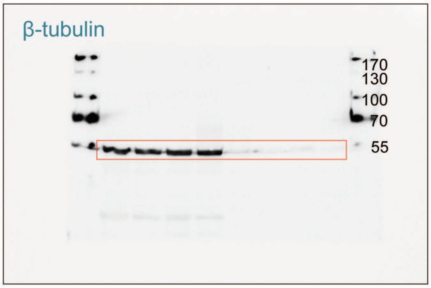

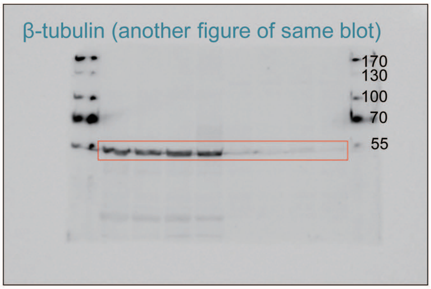

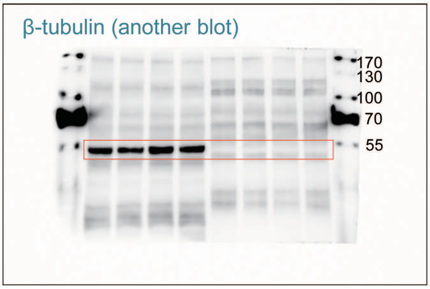

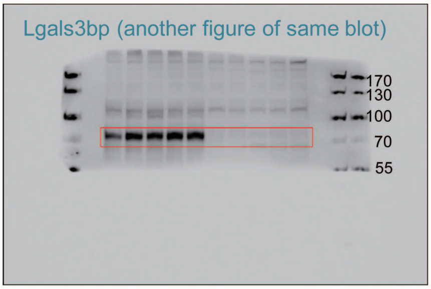

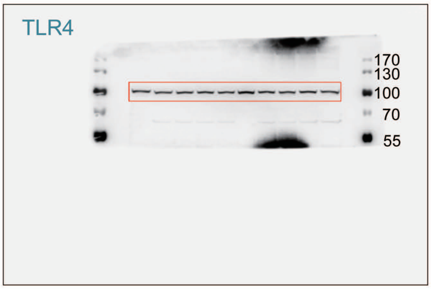

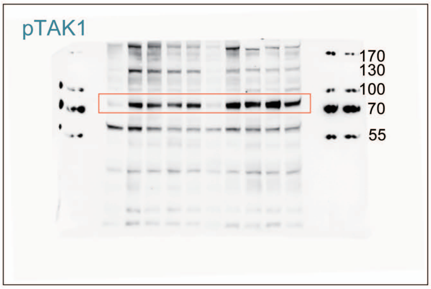

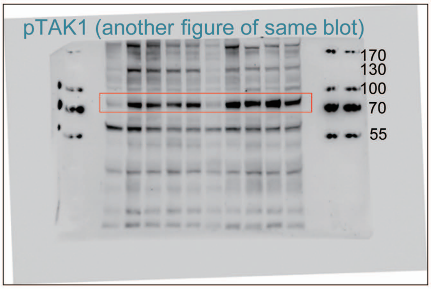

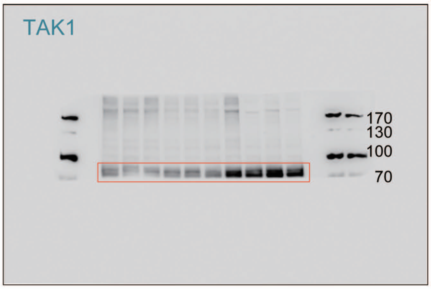

Fig 4E

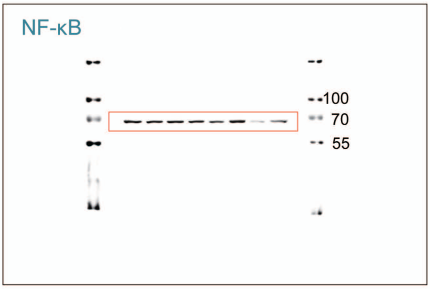

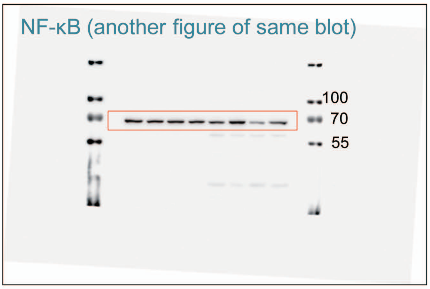

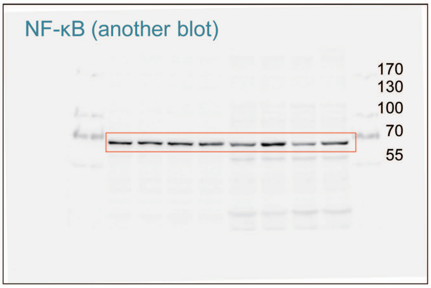

Fig 6A

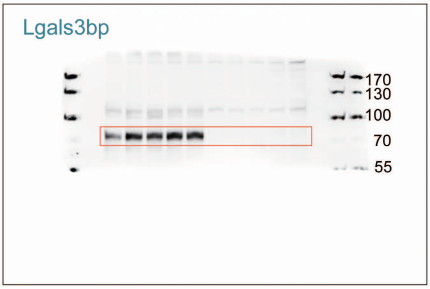

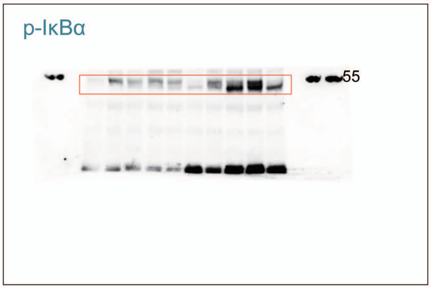

Fig 6A (continued)

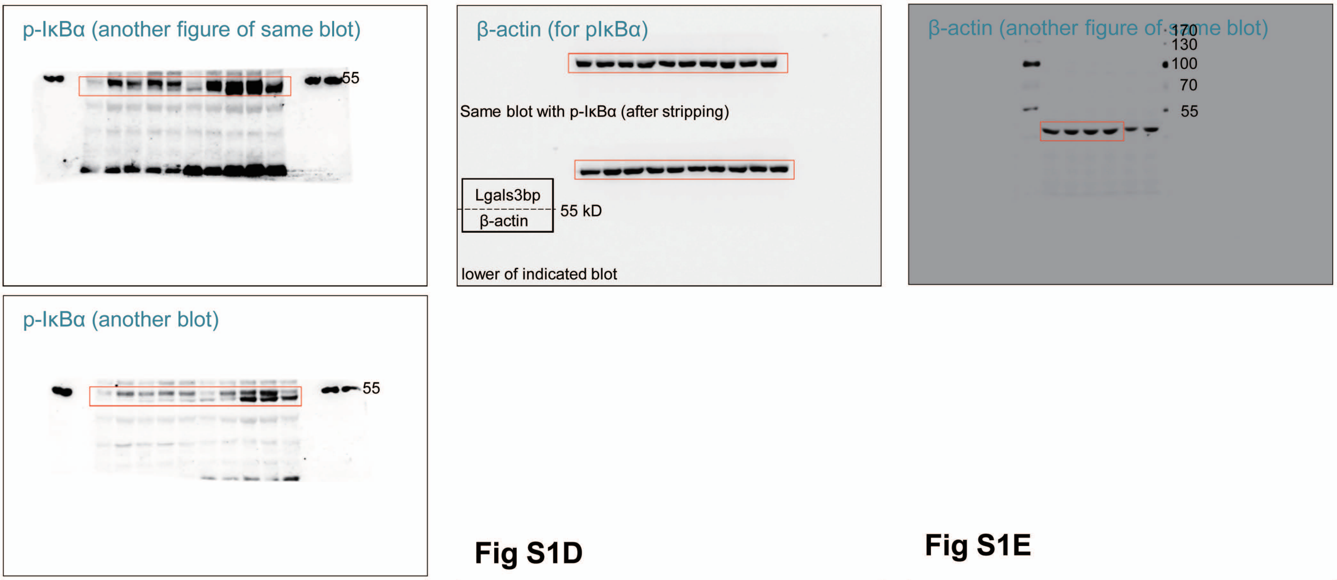

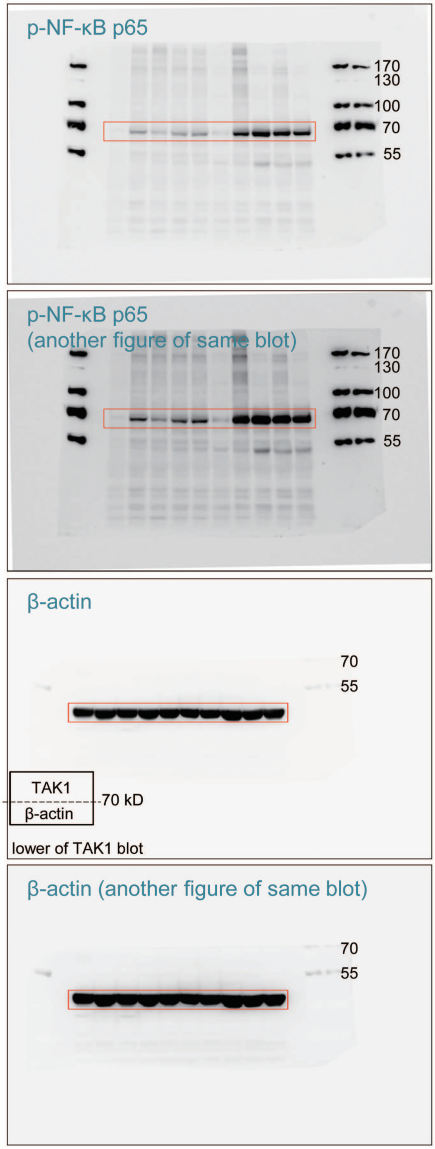

Fig S1D

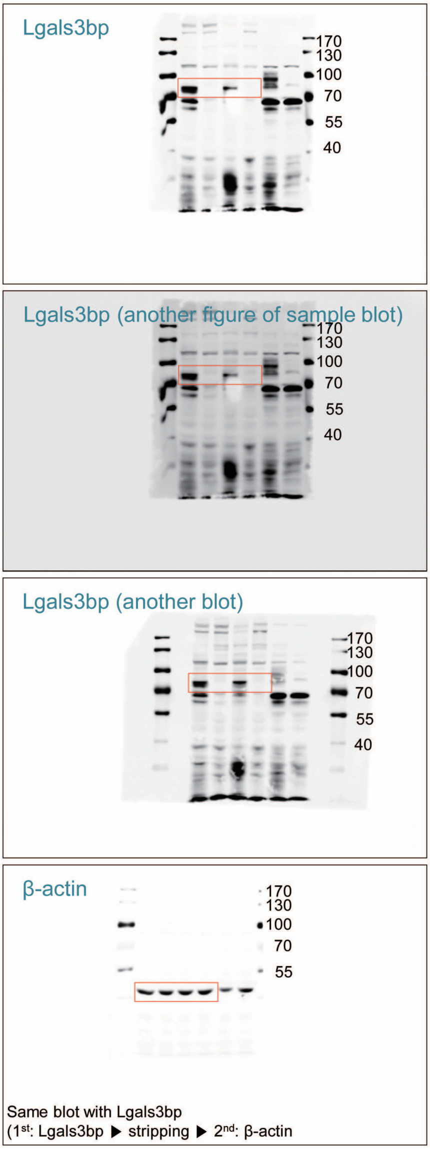

Fig S1E

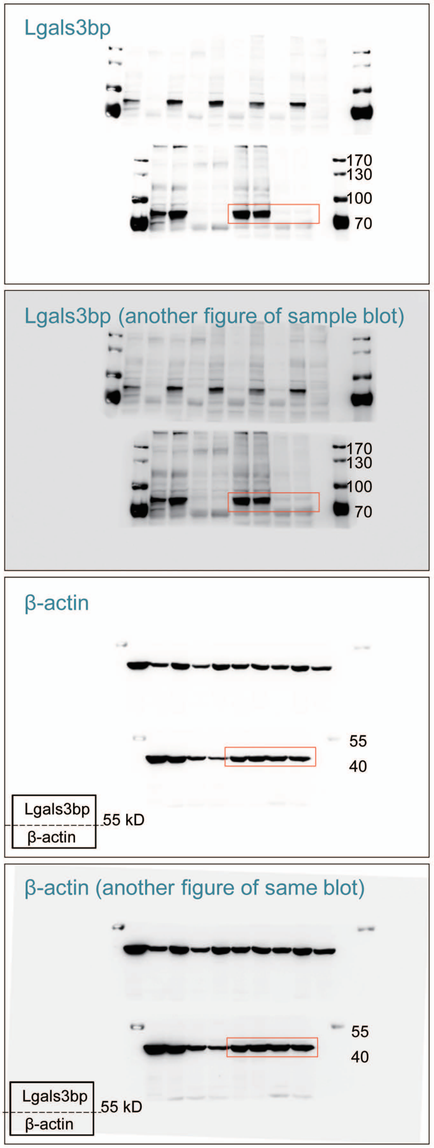

Supplement: Supplementary file 5 — Figure S3. [file 41420_2021_447_MOESM5_ESM.pdf]
